# Improving the Gel Quality of Threadfin Bream (*Nemipterus* spp.) Surimi Using Salted Duck Egg White Powder

**DOI:** 10.3390/foods11213350

**Published:** 2022-10-25

**Authors:** Naphat Wasinnitiwong, Samad Tavakoli, Soottawat Benjakul, Hui Hong

**Affiliations:** 1 Beijing Laboratory for Food Quality and Safety, College of Food Science and Nutritional Engineering, China Agricultural University, Beijing 100083, China; 2 International Center of Excellence in Seafood Science and Innovation, Faculty of Agro-Industry, Prince of Songkla University, Hat Yai 90110, Thailand; 3 Center of Food Colloids and Delivery for Functionality, College of Food Science and Nutritional Engineering, China Agricultural University, Beijing 100083, China

**Keywords:** surimi, gel properties, salted duck egg, egg white, threadfin bream

## Abstract

The effect of salted duck egg white powder (SDEWP) as a source of salt at different salt equivalent (SE) levels (0–2.5% SE) on gel qualities and texture properties of threadfin bream surimi was investigated. SDEWP possessed a high protein (64.59%) and salt (34.86%) concentration. The breaking force of surimi gel increased by the amount of SDEWP (*p* < 0.05). The addition of SDEWP up to 1.5% SE into the surimi gel has significantly increased the deformation (13.8%) and decreased the expressible moisture content (38.5%). Hardness, gumminess, and chewiness were augmented as higher levels of SDEWP were added, whereas cohesiveness decreased when the SDEWP above 1.5% SE was used. The incorporation of SDEWP had no significant effect on the springiness (*p* < 0.05). Less proteolytic degradation was observed in the surimi gel fortified with SDEWP. A higher amount of added SDEWP enhanced the whiteness of the surimi gel (*p* < 0.05). A denser and more ordered gel network was attained when SDEWP with 1.5% SE was added. SDEWP could be exploited as a source for salt and non-fish proteins in threadfin bream surimi, in which SDEWP containing 1.5% SE could significantly improve the quality of surimi gel.

## 1. Introduction

Surimi is a kind of processed fish made from deboned and minced fish meat. Surimi consists of concentrated fish myofibrillar proteins [1]. Production of a variety of food items with typical textures including fish balls, crab sticks, and fish tofu requires surimi as its raw material. The washing process is an essential step of surimi manufacturing which removes most of the water-soluble proteins and other impurities [2]. Gelation of surimi occurs when myofibrillar proteins, mainly myosin heavy chain (MHC), undergo unfolding, followed by aggregation, in which a three-dimensional protein network can be formed. Salt (2–3%) is usually added to surimi paste to enhance the solubilization or unfolding of MHC, thereby favoring surimi gel formation [3]. In general, heat-induced aggregation is a typical process for surimi gel production. Indigenous transglutaminase, which induces the formation of a non-disulfide covalent bond called gamma-Glu-epsilon-Lys isopeptides, is activated during a setting process. A setting process for surimi paste is commonly carried out at 40 °C [4,5,6], then cooked at 90 °C. To improve surimi gel properties, protein additives such as whey proteins, egg white proteins, and plasma proteins are usually incorporated into surimi [7]. These protein additives improve surimi gel quality by forming gel by themselves, as well as functioning as protease inhibitors [8,9]. Salted egg white is also a potential protein additive as it can increase gel strength and enhance the textural property of surimi gel made from sardine [10].

Salted egg, especially egg yolk, is a well-known preserved egg product. Various Chinese and Southeast Asian foods and desserts use salted egg yolk as their main ingredient. On the other hand, the salted egg white produced during salted egg yolk manufacturing is considered a by-product and is mostly discarded since it contains a large amount of salt and has a salty taste. Nevertheless, salted egg white is rich in proteins [11] and consists of 4–7% salt [12]. Proper disposal of salted egg white requires additional cost and improper disposal of the salted egg white may result in environmental pollution [13]. The exploitation of salted egg white can reduce the waste released into the environment. In addition, salted egg manufacturers can earn benefits, and low-value salted egg white can be better exploited.

The use of salted egg white could be maximized by utilizing salted egg white in surimi manufacturing as a source for salt and egg white proteins. The addition of salted duck egg white powder (SDEWP) might enhance the gel properties of surimi, as well as supply the needed salt, instead of using commercial salt. To the best of our knowledge, the effects of SDEWP on the gel properties of surimi from threadfin bream have not yet been studied. Threadfin bream is a tropical fish widely used for surimi production. Success in the application of salted egg white as a substitute for salt and egg white in surimi production may increase the market value of the useless salted egg white and reduce the cost required for disposing of it. Moreover, the utilization of salted egg white can prevent the wasting of a high-quality protein source and reduce the risk of water pollution from waste with high salt and protein concentration. This study aims to elucidate the impact of SDEWP on gel and texture properties of threadfin bream surimi when added at different SE levels.

## 2. Materials and Methods

### 2.1. Materials

Grade A frozen threadfin bream surimi was used in this study (Chaicharoen Marine (2002) Co., Ltd., Pattani, Thailand). Surimi consisted of 77.99% moisture, 13.03% protein, 3% sucrose, 3% sorbitol, and 0.50% polyphosphate (*w/w*). Salted duck eggs acquired from a local market in Hat Yai (Songkhla, Thailand) were used. Ethanol was purchased from RCI LABSCAN LTD. (Bangkok, Thailand). Chemicals supplied by Bio-Rad (Hercules, CA, USA) were used for sodium dodecyl sulfate-polyacrylamide gel electrophoresis. Other chemicals were obtained from LobaChemie Pvt. Ltd. (Mumbai, Maharashtra, India).

### 2.2. Preparation of Salted Duck Egg White Powder (SDEWP)

Firstly, egg white was collected from whole salted duck eggs. The egg white was then freeze-dried at −40 °C for 72 h using a freeze dryer (Scanvac CoolSafe 55, Labogene, Allerød, Denmark) and ground into powder. The egg white powder was places in a plastic zipper bag, and stored in a desiccator at room temperature to avoid moisture until used. The resulting powder was dubbed ‘SDEWP’.

### 2.3. Determination of Salt and Protein Contents of SDEWP

The salt content of SDEWP was determined using silver nitrate as per the AOAC method. The Kjeldahl method with a conversion factor of 6.25 was used for determining protein content [14]. Both salt and protein contents were reported as percentages (based on dry weight).

### 2.4. Preparation of Surimi Gel Added with SDEWP at Different SE Levels

The procedure of Buamard and Benjakul [15] was adopted with minor adjustments for preparing surimi gel. Frozen surimi was defrosted at 4 °C for 1 h. Surimi was chopped and added with SDEWP at different amounts possessing varying salt equivalent (SE) levels along with salt at the designated amount, as shown in Table 1. Salt equivalent level indicated the amount of salt added into surimi as a result of the addition of SDEWP. All samples had a final salt content of 2.5%. Iced water was added to the paste to adjust the final moisture content to 80%. A plastic casing with a 25 mm diameter was used for shaping surimi paste. The paste was incubated at 40 °C for 30 min and then boiled at 90 °C for 20 min. The gel was then immersed in ice for 1 h. Surimi gel samples added with 0%, 1%, 1.5%, 2%, and 2.5% SE of SDEWP were referred to as control, S1, S2, S3, and S4, respectively.

### 2.5. Determination of Properties of Surimi Gels

#### 2.5.1. Measurement of Dynamic Rheology of Surimi Paste

Surimi pastes containing salt and SDEWP at various SE (as shown in Table 1) were subjected to dynamic rheological measurement according to Singh and Benjakul [5] using a HAAKE RheoStress1 rheometer (ThemoFisher Scientific, Karlsruhe, Germany) with a Peltier temperature-controlled measuring plate. A 35 mm parallel plate geometry was used for measurement. The gap was set at 1 mm. The sample was heated at a heating rate of 1 °C/min from 10 to 90 °C. The measurement was conducted with 1 Hz oscillation and 1% deformation.

#### 2.5.2. Measurement of Breaking Force and Deformation

A puncture test of surimi gels was conducted using the method of Buamard and Benjakul [6]. A Model TA-XT2 texture analyzer (Stable MicroSystems, Surrey, UK) and a 5 mm spherical probe were used in this test. The result was recorded as breaking force (g) and deformation (mm). Before analysis, surimi samples were cut into cylinders (25 mm diameter and 25 mm height) and equilibrated at room temperature for 1 h.

#### 2.5.3. Determination of Texture Profile

Texture profile analysis (TPA) was conducted as described by Quan and Benjakul [10], using a Model TA-XT2i texture analyzer (Stable MicroSystem, Surrey, UK). The measurement was carried out using a 50 mm cylindrical aluminum probe. The measurement was conducted by compressing the cylindrical-shaped sample to 50% of its original height twice. The compressing and retracting speed was set as 5 mm/s. Hardness, springiness, cohesiveness, gumminess, and chewiness were recorded.

#### 2.5.4. Determination of Expressible Moisture Content

The method of Buamard et al. [6] was followed for the evaluation of expressible moisture content (EMC). The cylindrical-shaped sample was sliced into a 5 mm-thick piece and accurately weighed. This weight was referred to as X (g). The sample was placed on 3 pieces of filter paper and covered with 2 pieces of filter paper. A 5 kg standard weight was then put on top and held for 2 min. The sample was then accurately weighed again. This weight was referred to as Y (g). The equation shown below was used to calculate EMC.

EMC (%) = ((X − Y)/X) × 100

#### 2.5.5. Determination of Protein Pattern Using SDS-Polyacrylamide Gel Electrophoresis (SDS-PAGE)

Firstly, 27 mL of 5% SDS solution (95 °C) was added to surimi paste or gel sample (3 g) and homogenized using an IKA T25 digital ULTRA-TURRAX homogenizer (IKA-Werke GmbH & Co. KG, Staufen, Germany) at 6000 rpm to solubilize the samples. To dissolve the sample, the homogenate was then incubated for 60 min at 95 °C. The protein sample solution was separated from undissolved matter by centrifuging using a Model Allegra 25R centrifuge (Beckman Coulter, Palo Alto, CA, USA) at 6050× *g* for 20 min at 25 °C. A biuret test was conducted to determine the protein concentration of the protein sample [16]. SDS-PAGE was performed as described by Laemmli [17]. SDS-PAGE consisted of 10% running gel and 4% stacking gel was used. A Miniprotein II electrophoresis unit (Bio-Rad Laboratories, Richmond, CA, USA) was used for electrophoresis at a constant current of 15 mA/gel. After separation, the procedure outlined by Quan and Benjakul [10] was adopted for staining and destaining the protein bands.

#### 2.5.6. Determination of Whiteness

The whiteness of surimi gel samples was measured using a colorimeter (ColorFlex EZ, Hunter Lab Reston, Reston, VA, USA). The whiteness of the surimi gel was calculated from the measured L*, a*, and b* values [6].

Whiteness = 100 − ((100 − *L*)^2^ + a*^2^ + b*^2^)^1/2^

#### 2.5.7. Determination of TCA-Soluble Peptide Content

The surimi gel sample (3 g) was solubilized by adding 27 mL of 5% TCA (4 °C) and homogenized at 6000 rpm. The solubilized sample was then put at 4 °C for 8 h. Next, the undissolved matter was removed from the mixture by centrifuging at 6050× *g* for 20 min at 25 °C using a Model Allegra 25R centrifuge (Beckman Coulter, Palo Alto, CA, USA). The TCA-soluble peptide content of the sample was measured from the collected supernatant with the Lowry method [18], and calculated as μmol tyrosine equivalent/g sample.

#### 2.5.8. Microstructure

A scanning electron microscope (Quanta 400-FEG SEM, FEI company, Hillsboro, OR, USA) was used for visualizing the microstructure of surimi gel samples (×10,000 magnification). A sample for SEM scanning was prepared as detailed by Buamard et al. [6]. Firstly, cube-shaped surimi gels with a thickness of 2–3 mm were prepared. The sample was then immersed in 3.0% (*v/v*) glutaraldehyde in 0.2 M phosphate buffer (pH 7.2) for 3 h at room temperature. The sample was washed with distilled water and dehydrated by immersing it in ethanol with serial concentrations of 25%, 50%, 70%, 80%, and 100%. The sample was critical point dried with CO_2_ as a transition fluid and sputter-coated with gold.

### 2.6. Statistical Analysis

SPSS version 17.0 (SPSS, Inc., Chicago, IL, USA) was used for performing statistical analysis. All data were subjected to a one-way analysis of variance (ANOVA). Mean comparison was carried out using Duncan’s multiple range test at a level of *p* < 0.05.

## 3. Results and Discussion

### 3.1. Dynamic Rheology of Threadfin Bream Surimi Pastes Containing SDEWP at Various SE Levels

**Figure 1:**
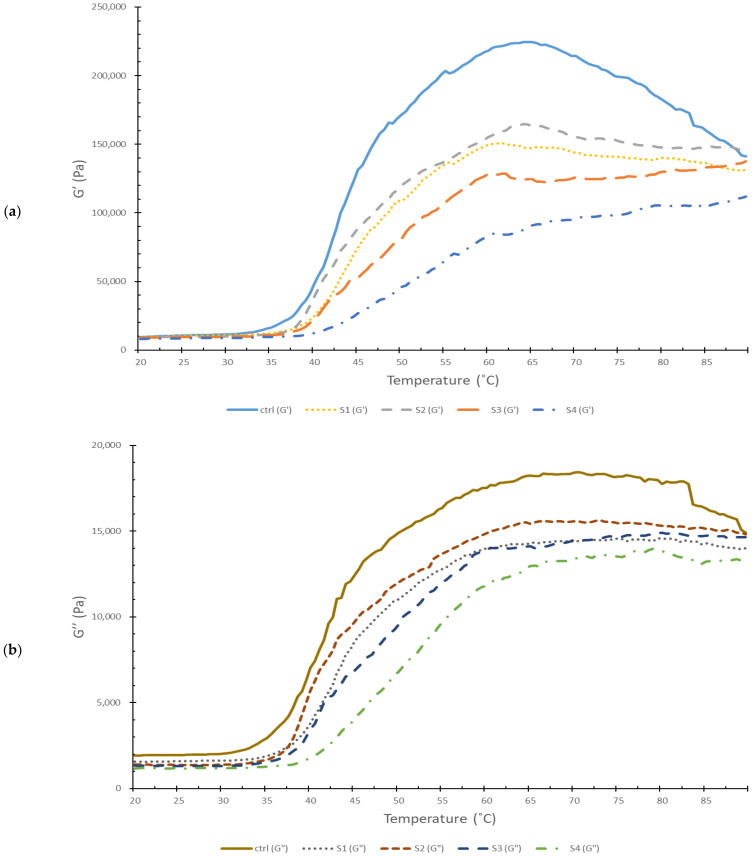
(**a**) Storage moduli (G’) and (**b**) loss modulus (G”) of threadfin bream surimi gels added with SDEWP at 0% (ctrl), 1% (S1), 1.5% (S2), 2% (S3), and 2.5% (S4) salt equivalent levels.

The transition of storage modulus (G’) and loss modulus (G”) of surimi pastes from threadfin bream added with different SE levels of SDEWP was investigated (Figure 1a,b). All samples expressed a similar pattern in the G’ graph as the temperature raised from 20 °C to 90 °C. G’ value is a parameter representing the elastic behavior of the sample when subjected to shear force, while G” represents the viscous behavior of the sample [19]. The much higher G’ than G” observed in all samples throughout the test indicated that the samples’ behavior was more solid-like [19]. As the temperature increased, myofibrillar proteins denatured, cross-linked, and aggregated, and resulted in changes in surimi gel properties. This phenomenon was represented by the G’ values. The G’ curve of all samples remained constant up to approximately 35 °C. This pattern of the G’ curve was also observed in surimi made from gurnard [20]. Subsequently, the G’ value of all samples started rising at different rates. Heavy meromyosin unfolds at this temperature range and releases myosin filaments. The cross-linking of these myosin filaments increased the G’ value [21]. At high temperatures, proteins underwent unfolding and entanglement or aggregation took place easily. Different patterns of the G’ graph in different samples probably resulted from the varying amounts of SDEWP added. Protein in SDEWP could dilute myofibrillar proteins in surimi. Consequently, the formation of a strong myofibril network was lowered, especially when the SDEWP amount increased. This was justified by the lower G’. In addition, Li et al. [22] documented that G’ of egg white continuously decreased during the 25 °C to 65 °C temperature range. The main protein components of egg white are 54% ovalbumin and 12% conalbumin. These proteins started gelling at 75 °C and 62 °C, respectively [23]. After the temperature was above 60–65 °C, the G’ of the control sample drastically dropped. Wijayanti et al. [24] reported that regression of Gʹ at around 70 °C was observed in threadfin bream surimi added with bio-calcium. A drop in G’ was plausibly caused by losing hydrogen bonds under high temperatures [25]. Samples added with SDEWP at 1.0 and 1.5% SE (S1 and S2) had a slight decrease up to 80 °C, while those incorporated with SDEWP at 2.0 and 2.5% SE (S3 and S4) possessed the constant G’, regardless of temperatures. The unchanged Gʹ value after 60 °C in samples with SDEWP addition at high levels might be caused by the gelation of egg white proteins as indicated by the rapidly increased G’ value of egg white after 60 °C [22]. At the final temperature, it was observed that samples added with SDEWP at 1.5% SE (S2) showed the highest G’. This indicated the higher conversion of liquid to the solid state of surimi paste, reflecting the stronger gel stabilized by several bonds.

### 3.2. Breaking Forces and Deformations

The gel strength of surimi is determined by the firmness and elasticity of the gel, which is represented by breaking force and deformation, respectively [26]. Surimi gel samples containing SDEWP have a greater breaking force value than the control (Table 2). The increase occurred at a greater magnitude with increasing SDEWP levels. Quan and Benjakul [10] documented that the breaking force of surimi gel made from sardine can be enhanced by adding salted egg albumen. The breaking force reached the maximum value when SDEWP was added at 2.5% SE. The increase was 78.3% compared to that of the control. The differences in breaking force values of the samples containing SDEWP at 1–2% SE (S1–S3) were not significant (*p* < 0.05). The addition of SDEWP at 1–1.5% increased the deformation of surimi gel up to 13.8% from that of the control sample. However, around a 6.3% reduction of deformation value was gained compared to that of the control when SDEWP above 2.0% SE was used. The coagulum gel formed as egg white proteins undergoing heat-induced gelation could reinforce surimi gel by filling the voids in the gel structure, thus increasing the surimi gel sample’s breaking force. In surimi products, egg white proteins commonly serve as a gelling agent [23]. It can be used as a protein additive for improving the gel strength of threadfin bream surimi [27]. However, the gelation of myofibrillar proteins can be interfered with by egg white proteins and results in surimi gel with less strength when egg white proteins are added in excessive amounts [28]. This might result in decreased deformation when SDEWP at higher amounts was incorporated. Proteins in SDEWP might have lower gel-forming ability than normal egg white [13] and negatively affect surimi gel strength when added in a large amount. This result identified the potential of SDEWP as a source of salt in surimi manufacturing. Egg white protein present in SDEWP also improved surimi gel strength. However, an excessive amount of SDEWP can reduce the elasticity of surimi gel from threadfin bream.

### 3.3. Expressible Moisture Contents (EMC)

All surimi samples incorporated with SDEWP have reduced expressible moisture content (EMC) compared to the control, as shown in Table 2. The lowest EMC was attained from the surimi gel sample containing 1.5% SE (S2), in which EMC was 38.5% lower than that of the control sample. A lower EMC value indicated higher water holding capacity (WHC) of surimi gel as the gel could release less water and trap more water inside its gel network [29]. Surimi made from lizardfish, goatfish, bigeye snapper, Indian mackerel, and grass carp showed increased WHC when egg white proteins were added to the gel [5,27,30]. Both myofibrillar proteins and egg white proteins could form a strong gel network, which could entrap more water inside. SDEWP at the appropriate level (up to 1.5% SE) could thus improve the WHC of the surimi gel.

### 3.4. Texture Profiles

Texture profiles of surimi gel samples containing SDEWP at different amounts are shown in Table 3. The addition of SDEWP resulted in higher hardness, gumminess, and chewiness in threadfin bream surimi gel (*p* < 0.05), especially at a high SE level. No difference in the three parameters was observed between S1 and S2 samples (*p* < 0.05). Additionally, no difference in those parameters between S3 and S4 samples was observed (*p* < 0.05) either. The maximum increase in hardness, gumminess, and chewiness value of 86.1%, 80.5%, and 78%, respectively, was obtained from the gel containing SDEWP at 2.5% SE (S4). An increase in hardness of the samples containing SDEWP with high SE levels was agreed with their increased breaking force. On the other hand, the cohesiveness of the surimi gel remained constant when surimi gel samples contained less than 1.5% SE (*p* < 0.05) of SDEWP, whereas the regress was found in the samples containing more than 2% SE of SDEWP (*p* < 0.05). This pattern of cohesiveness value implied that proteins in SDEWP might disrupt the aggregation of myofibrillar proteins and lead to a decrease in elasticity. The addition of SDEWP did not significantly affect the springiness of surimi gel regardless of the amount added (*p* < 0.05). Upsurges in hardness, gumminess, and cohesiveness of surimi gel containing SDEWP were also reported by Quan and Benjakul [10]. Hardness is a force required for deforming the sample [31]. The addition of SDEWP enhanced surimi gel hardness as egg white proteins can form a heat-induced gel with good gel texture [23]. Moreover, egg white proteins also protected MHC from protease associated with muscle proteins by acting as protease inhibitors [32]. Gumminess and chewiness similarly increased to hardness value as the two parameters were calculated from hardness value [11]. Cohesiveness is a parameter representing the force required to overcome internal bonding [31]. The fortification of egg white protein into the surimi of Alaska pollock and Pacific whiting did not improve their cohesiveness [33]. The gel from salted egg white proteins had significantly lower cohesiveness than normal egg white [11]. Thus, the textural property of surimi gel from threadfin bream can be altered with the addition of SDEWP. Its impact was dependent upon the amount added.

### 3.5. Protein Patterns of Surimi Pastes and Gels Containing SDEWP at Different SEs

**Figure 2:**
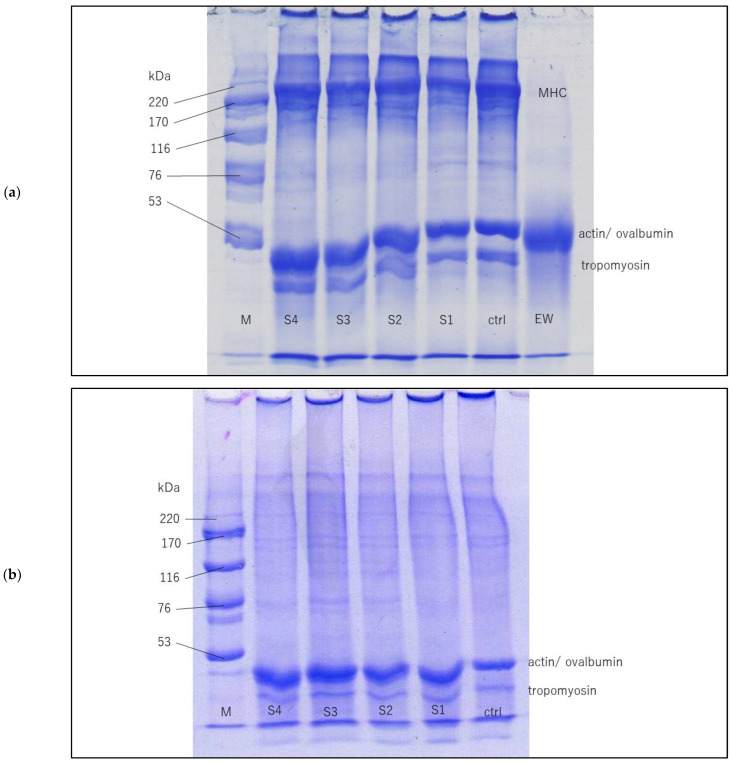
Protein pattern of threadfin bream surimi paste (**a**) and gel (**b**) samples added with SDEWP at 0% (ctrl), 1% (S1), 1.5% (S2), 2% (S3), and 2.5% (S4) salt equivalent levels. MHC: myosin heavy chain, M: high molecular weight marker.

Protein patterns of surimi pastes and gels added with SDEWP at varying amounts are shown in Figure 2. Myosin heavy chain (MHC), actin, and tropomyosin were found as major proteins in threadfin bream surimi. MHC band in the paste without SDEWP (control) appeared with slightly higher intensity than those containing SDEWP. The lower band intensity was likely caused by lower MHC concentration in the surimi sample containing SDEWP. The band at 45 kDa was increased in intensity for the paste sample added with higher levels of SDEWP. Egg white composes mainly of ovalbumin, which has a similar molecular weight (MW) of 45 kDa [8]. Therefore, the increase in MW at 45 kDa, especially for S4, which contained SDEWP at 2.5% SE was attributed to the increase in ovalbumin from SDEWP. No marked difference was found in the intensity of the tropomyosin band among all the paste samples. 

The pattern of actin/ovalbumin bands and tropomyosin bands of each surimi gel sample was similar to that of the corresponding paste sample. However, no MHC band appeared in the protein pattern of any surimi gel sample. The formation of non-sulfide covalent bonds which plays an important role in the cross-linking of MHC was most likely responsible for this change. The non-sulfide covalent bond, which is induced by transglutaminase during the setting process, cannot be cleaved by either SDS (cleaves non-covalent bond) or 2-mercaptoethanol (cleaves disulfide bond) contained in the sample buffer. Thus, the high molecular weight protein aggregates formed during the setting process remain intact and retained in the wells as they could not pass through the stacking gel [30]. This resulted in the disappearance of the MHC band in every gel sample’s protein pattern. Additionally, SDEWP had no profound interfering effect on MHC polymerization during the thermal gelling process.

### 3.6. Color

L*, a*, and b* values recorded from gel samples containing different SE levels of SDEWP were used for the calculation of the gel’s whiteness (Table 4). A slightly greater L* value than the Ctrl was found when SDEWP, especially at higher SE levels, was added (*p* < 0.05). This coincided with a greater whiteness value for samples containing SDEWP. The a* and b* values of all surimi samples were not significantly different regardless of the amount of SDEWP added (*p* < 0.05). Jafarpour et al. [9] also reported that L* and whiteness of surimi gel from common carp increased when egg white was added. The increase in the L* value of surimi samples containing SDEWP is likely caused by the white color of SDEWP. The color of surimi gel can be altered by the color of the additives used [34]. Generally, high L* and whiteness values are considered desirable characteristics of surimi [20]. This result indicated that using SDEWP as a source of salt in surimi helped enhance the whiteness of threadfin bream surimi gel.

### 3.7. TCA-Soluble Peptide Content (TCA-SPC)

TCA-SPCs of threadfin bream surimi gel samples containing different SE levels of SDEWP are shown in Table 2. TCA-SPC represents the proteolysis of myofibrillar proteins in surimi that occurred when surimi is subjected to the setting and heating process [6]. Samples containing SDEWP expressed lower TCS-SPC (*p* < 0.05). However, the amount of SDEWP added did not cause any significant difference in TCS-SPC among the samples with SDEWP. The reduced TCA-soluble peptides in surimi samples containing SDEWP were likely caused by the inhibitory activity of egg white protein from SDEWP. Egg white contains several proteins that act as protease inhibitors. Serine proteases can be inhibited by ovomucoid, ovomacroglobulin, and other ovoinhibitors found in egg white [35]. Both serine protease and metalloprotease can be inhibited by ovostatin from duck eggs [36]. Sardine surimi added with SDEWP also showed a reduced TCA-SPC [10]. This result suggested that proteolytic degradation in threadfin bream surimi mediated by indigenous proteases can be prevented using SDEWP.

### 3.8. Microstructure 

**Figure 3:**
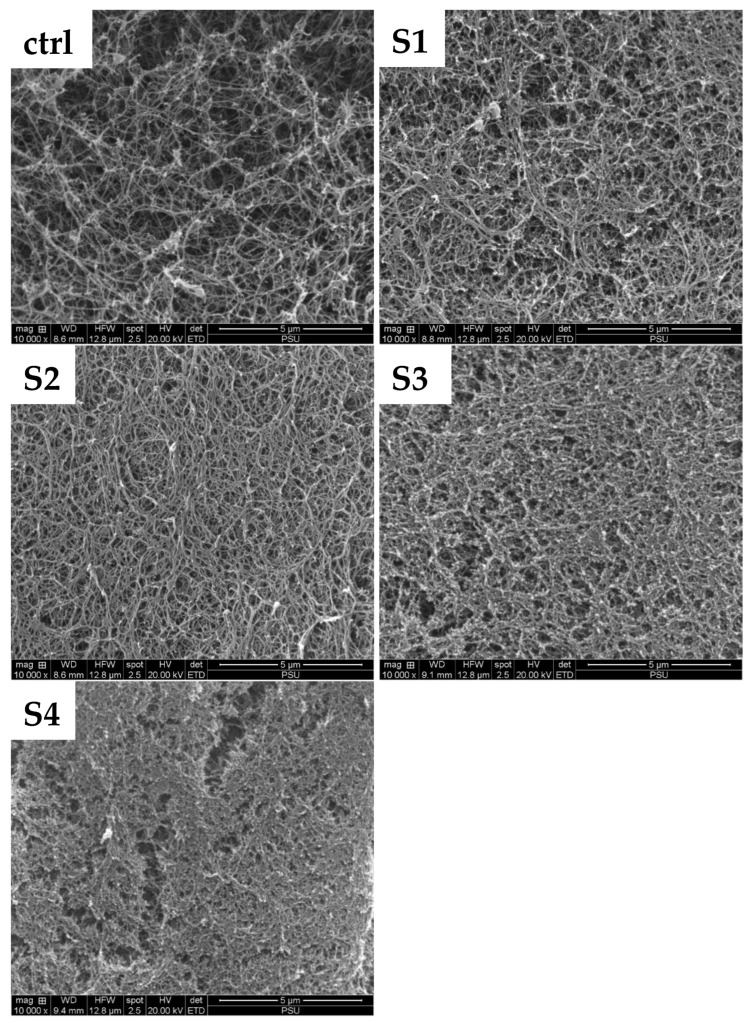
Microstructure of surimi gels added with SDEWP at 0% (ctrl), 1% (S1), 1.5% (S2), 2% (S3), and 2.5% (S4) salt equivalent levels (magnification ×10,000).

Surimi gels fortified with SDEWP presented a more packed and finer structure than the control sample (Figure 3). The greater breaking force (Table 2) and hardness (Table 3) values observed were in line with this finding. However, small cavities appeared in S3 and S4 samples, containing 2% and 2.5% SE of SDEWP, respectively. This was coincidental with decreased deformation (Table 2) and cohesiveness (Table 3) and increased EMC (Table 2). SDEWP contains a large quantity of egg white protein, which acts as a protease inhibitor and filler [23,35,36] in surimi products. A denser and more ordered gel structure mediated by connected protein strands was achieved when SDEWP at an appropriate level was added. Nevertheless, an excessive amount of SDEWP added could dilute myofibrillar proteins in surimi, resulting in a brittle gel as witnessed by the loss in elasticity. Surimi gel with less elasticity is less desirable for customers. Thus, SDEWP influenced the microstructure of the gel network of surimi from threadfin bream.

## 4. Conclusions

Salted duck egg white powder containing high salt and protein content could be used as an alternative additive in surimi. Egg white proteins contained in salted duck egg white more likely worked as gelling protein and protease inhibitors. The addition of SDEWP at 1.5% SE improved the gel strength, WHC, and textural quality of surimi gel made from threadfin bream. In addition, a denser and more ordered gel network was formed in surimi gel added with SDEWP at 1.5% SE. Therefore, SDEWP could be exploited as a potential additive in surimi manufacturing.

## Figures and Tables

**Table 1:** Amount of salt and salted duck egg white powder (SDEWP) used in surimi.

Treatments	Salt Added (g)	SDEWP Added (g)	Salt Equivalent in SDEWP (g)	Protein in SDEWP (g)
ctrl	2.5	0.00	0.0	0.00
S1	2.0	3.16	1.0	2.04
S2	1.5	4.74	1.5	3.06
S3	1.0	6.31	2.0	4.08
S4	0.0	7.89	2.5	5.10

Surimi of 100 g was used. SDEWP consisted of 64.59 ± 0.96% protein and 34.86 ± 1.39% salt (dry basis).

**Table 2:** Breaking force, deformation, expressible moisture content, and TCA soluble peptide content of threadfin bream surimi containing SDEWP at 0% (ctrl), 1% (S1), 1.5% (S2), 2% (S3), and 2.5% (S4) SE levels.

Samples	Breaking Force (g)	Deformation (mm)	EMC (%)	TCA-Soluble Peptide Content (μmol Tyrosine Equivalent/g Sample)
ctrl	358.23 ± 7.07 c	7.51 ± 0.13 b	4.71 ± 0.17 e	14.39 ± 0.37 a
S1	576.92 ± 8.53 b	8.55 ± 0.29 a	3.48 ± 0.12 b	12.57 ± 0.43 b
S2	593.97 ± 17.66 b	8.29 ± 0.44 a	2.97 ± 0.14 a	13.27 ± 0.03 b
S3	595.46 ± 20.226 b	7.12 ± 0.08 c	3.75 ± 0.08 c	12.58 ± 0.13 b
S4	638.87 ± 4.44 a	7.04 ± 0.04 c	4.30 ± 0.12 d	12.97 ± 0.56 b

SDEWP, salted duck egg white powder; SE, salt equivalent. Different lowercase letters in the same column indicate significant differences (*p* < 0.05).

**Table 3:** Texture profile of threadfin bream surimi containing SDEWP at 0% (ctrl), 1% (S1), 1.5% (S2), 2% (S3), and 2.5% (S4) SE levels.

Samples	Hardness (N)	Springiness (cm)	Cohesiveness	Gumminess (N)	Chewiness (N.cm)
ctrl	76.28 ± 3.11 c	0.917 ± 0.01 ab	0.771 ± 0.00 a	58.77 ± 2.11 c	53.86 ± 1.36 c
S1	108.01 ± 1.66 b	0.930 ± 0.01 a	0.773 ± 0.00 a	83.47 ± 1.28 b	77.65 ± 1.08 b
S2	115.24 ± 2.50 b	0.907 ± 0.01 b	0.769 ± 0.00 a	88.64 ± 1.45 b	80.44 ± 0.57 b
S3	143.02 ± 12.96 a	0.907 ± 0.00 b	0.744 ± 0.01 b	106.36 ± 8.12 a	96.45 ± 7.24 a
S4	141.99 ± 3.04 a	0.903 ± 0.01 b	0.747 ± 0.00 b	106.08 ± 1.97 a	95.81 ± 1.20 a

SDEWP, salted duck egg white powder; SE, salt equivalent. Different lowercase letters in the same column indicate significant differences (*p*  <  0.05).

**Table 4:** L*, a*, b* and whiteness of threadfin bream surimi containing SDEWP at 0% (ctrl), 1% (S1), 1.5% (S2), 2% (S3), and 2.5% (S4) SE levels.

Samples	L*	a*	b*	Whiteness
ctrl	78.05 ± 0.44 c	0.76 ± 0.04 a	14.00 ± 0.48 a	73.95 ± 0.50 b
S1	78.31 ± 0.23 c	0.77 ± 0.05 a	13.83 ± 0.37 a	74.27 ± 0.31 b
S2	78.55 ± 0.28 bc	0.73 ± 0.02 a	13.76 ± 0.26 a	74.50 ± 0.16 ab
S3	78.96 ± 0.34 ab	0.73 ± 0.06 a	13.68 ± 0.45 a	74.96 ± 0.42 a
S4	79.12 ± 0.71 a	0.75 ± 0.04 a	13.55 ± 0.45 a	75.07 ± 0.65 a

SDEWP, salted duck egg white powder; SE, salt equivalent. Different lowercase letters in the same column indicate significant differences (*p*  <  0.05).

## Data Availability

Data is contained within the article or supplementary material.

## References

[1] Yingchutrakul M., Wasinnitiwong N., Benjakul S., Singh A., Zheng Y., Mubango E., Luo Y., Tan Y., Hong H. (2022). Asian Carp, an Alternative Material for Surimi Production: Progress and Future. Foods.

[2] Nopianti R., Huda N., Ismail N. (2011). A Review on the Loss of the Functional Properties of Proteins during Frozen Storage and the Improvement of Gel-forming Properties. Am. J. Food Technol..

[3] Núñez-Flores R., Cando D., Borderías A.J., Moreno H.M. (2018). Importance of salt and temperature in myosin polymerization during surimi gelation. Food Chem..

[4] Rawdkuen S., Benjakul S. (2008). Whey protein concentrate: Autolysis inhibition and effects on the gel properties of surimi prepared from tropical fish. Food Chem..

[5] Singh A., Benjakul S. (2017). Effect of serine protease inhibitor from squid ovary on gel properties of surimi from Indian mackerel. J. Texture Stud..

[6] Buamard N., Benjakul S., Konno K. (2017). Improvement of gel quality of sardine surimi with low setting phenomenon by ethanolic coconut husk extract. J. Texture Stud..

[7] Walayat N., Xiong H., Xiong Z., Moreno H.M., Nawaz A., Niaz N., Randhawa M.A. (2020). Role of cryoprotectants in surimi and factors affecting surimi gel properties: A review. Food Rev. Int..

[8] Quan T.H., Benjakul S. (2017). Comparative study on the effect of duck and hen egg albumens on proteolysis and gel property of sardine surimi. Int. J. Food Prop..

[9] Jafarpour A., Hajiduon H.-A., Aie M.R. (2012). A comparative study on effect of egg white, soy protein isolate and potato starch on functional properties of common carp (*Cyprinus carpio*) surimi gel. J. Food Process. Technol..

[10] Quan T.H., Benjakul S. (2019). Impact of salted duck egg albumen powder on proteolysis and gelling properties of sardine surimi. J. Texture Stud..

[11] Quan T.H., Benjakul S. (2018). Compositions, protease inhibitor and gelling property of duck egg albumen as affected by salting. Korean J. Food Sci. Anim. Resour..

[12] Kaewmanee T., Benjakul S., Visessanguan W. (2009). Changes in chemical composition, physical properties and microstructure of duck egg as influenced by salting. Food Chem..

[13] Tang H., Tan L., Chen Y., Zhang J., Li H., Chen L. (2021). Effect of κ-carrageenan addition on protein structure and gel properties of salted duck egg white. J. Sci. Food Agric..

[14] Latimer G. (2012). Official Methods of Analysis of AOAC International.

[15] Buamard N., Benjakul S. (2015). Improvement of gel properties of sardine (*Sardinella albella*) surimi using coconut husk extracts. Food Hydrocoll..

[16] Kingsley G.R. (1939). The determination of serum total protein, albumin, and globulin by the biuret reaction. J. Biol. Chem..

[17] Laemmli U.K. (1970). Cleavage of structural proteins during the assembly of the head of bacteriophage T4. Nature.

[18] Lowry O.H., Rosebrough N.J., Farr A.L., Randall R.J. (1951). Protein measurement with the Folin phenol reagent. J. Biol. Chem..

[19] Tabilo-Munizaga G., Barbosa-Cánovas G.V. (2005). Rheology for the food industry. J. Food Eng..

[20] Zhou X., Chen T., Lin H., Chen H., Liu J., Lyu F., Ding Y. (2019). Physicochemical properties and microstructure of surimi treated with egg white modified by tea polyphenols. Food Hydrocoll..

[21] Chanarat S., Benjakul S., Xiong Y.L. (2015). Physicochemical changes of myosin and gelling properties of washed tilapia mince as influenced by oxidative stress and microbial transglutaminase. J. Food Sci. Technol..

[22] Li J., Zhang Y., Fan Q., Teng C., Xie W., Shi Y., Su Y., Yang Y. (2018). Combination effects of NaOH and NaCl on the rheology and gel characteristics of hen egg white proteins. Food Chem..

[23] Park J.W., Ooizumi T., Hunt A.L. (2013). Ingredient Technology for Surimi and Surimi Seafood. Surimi Surimi Seaf..

[24] Wijayanti I., Singh A., Benjakul S., Sookchoo P. (2021). Textural, sensory, and chemical characteristic of threadfin bream (*Nemipterus* sp.) surimi gel fortified with bio-calcium from bone of asian sea bass (*Lates calcarifer*). Foods.

[25] Lanier T.C., Carvajal P., Yongsawatdigul J. (2000). Surimi gelation chemistry. Surimi Surimi Seaf..

[26] Lin X., Yang W., Xu D., Jie Z., Liu W. (2015). Improving gel properties of hairtail surimi by electron irradiation. Radiat. Phys. Chem..

[27] Tadpitchayangkoon P., Park J.W., Yongsawatdigul J. (2012). Gelation characteristics of tropical surimi under water bath and ohmic heating. LWT-Food Sci. Technol..

[28] Sun X.D., Holley R.A. (2011). Factors influencing gel formation by myofibrillar proteins in muscle foods. Compr. Rev. Food Sci. Food Saf..

[29] Klomklao S., Benjakul S., Kishimura H., Osako K., Simpson B.K. (2016). Trypsin inhibitor from yellowfin tuna (*Thunnus albacores*) roe: Effects on gel properties of surimi from bigeye snapper (*Priacanthus macracanthus*). LWT.

[30] Yu X., Wang Y., Xie Y., Wei S., Ding H., Yu C., Dong X. (2022). Gelation properties and protein conformation of grass carp fish ball as influenced by egg white protein. J. Texture Stud..

[31] Liu Y., Sun Q., Pan Y., Wei S., Xia Q., Liu S., Ji H., Deng C., Hao J. (2021). Investigation on the correlation between changes in water and texture properties during the processing of surimi from golden pompano (*Trachinotus ovatus*). J. Food Sci..

[32] Singh A., Benjakul S. (2018). Proteolysis and its control using protease inhibitors in fish and fish products: A review. Compr. Rev. Food Sci. Food Saf..

[33] Tabilo-Munizaga G., Barbosa-Cánovas G.V. (2004). Color and textural parameters of pressurized and heat-treated surimi gels as affected by potato starch and egg white. Food Res. Int..

[34] Duangmal K., Taluengphol A. (2010). Effect of protein additives, sodium ascorbate, and microbial transglutaminase on the texture and colour of red tilapia surimi gel. Int. J. Food Sci. Technol..

[35] Solanki J., Zofair S., Parmar L., Ashok D., Kotia A., Gunalan B. (2011). Effect of egg albumen (protein additive) on surimi prepared from lizardfish (*Saurida tumbil*) during frozen storage. Aquac. Aquar. Conserv. Legis..

[36] Hu S., Qiu N., Liu Y., Zhao H., Gao D., Song R., Ma M. (2016). Identification and comparative proteomic study of quail and duck egg white protein using 2-dimensional gel electrophoresis and matrix-assisted laser desorption/ionization time-of-flight tandem mass spectrometry analysis. Poult. Sci..

